# Diurnal and Seasonal Variations in the Photosynthetic Characteristics and the Gas Exchange Simulations of Two Rice Cultivars Grown at Ambient and Elevated CO_2_

**DOI:** 10.3389/fpls.2021.651606

**Published:** 2021-04-06

**Authors:** Yuxuan Miao, Yao Cai, Hao Wu, Dan Wang

**Affiliations:** Department of Ecology, College of Applied Meteorology, Nanjing University of Information Science and Technology, Nanjing, China

**Keywords:** global change, photosynthesis, stomatal conductance, chlorophyll *a* fluorescence, photosynthesis model, stomatal conductance model

## Abstract

Investigating the diurnal and seasonal variations of plant photosynthetic performance under future atmospheric CO_2_ conditions is essential for understanding plant adaptation to global change and for estimating parameters of ecophysiological models. In this study, diurnal changes of net photosynthetic rate (A_net_), stomatal conductance (g_s_), and photochemical efficiency of PSII (F_v_′/F_*m*_′) were measured in two rice cultivars grown in the open-top-chambers at ambient (∼450 μmol mol^–1^) and elevated (∼650 μmol mol^–1^) CO_2_ concentration [(CO_2_)] throughout the growing season for 2 years. The results showed that elevated (CO_2_) greatly increased A_net_, especially at jointing stage. This stimulation was acclimated with the advance of growing season and was not affected by either stomatal limitations or Rubisco activity. Model parameters in photosynthesis model (V_cmax_, J_max_, and R_d_) and two stomatal conductance models (m and g_1_) varied across growing stages and m and g_1_ also varied across (CO_2_) treatments and cultivars, which led to more accurate photosynthesis and stomatal conductance simulations when using these cultivar-, CO_2_-, and stage- specific parameters. The results in the study suggested that further research is still needed to investigate the dominant factors contributing to the acclimation of photosynthetic capacity under future elevated CO_2_ conditions. The study also highlighted the need of investigating the impact of other environmental, such as nitrogen and O_3_, and non-environmental factors, such as additional rice cultivars, on the variations of these parameters in photosynthesis and stomatal conductance models and their further impacts on simulations in large scale carbon and water cycles.

## Introduction

Under the influence of human activities, atmospheric CO_2_ concentration [(CO_2_)] has been increasing to 415 μmol mol^–1^ in 2019 since the Industrial Revolution, before which the (CO_2_) was steadily maintained at about 280 μmol mol^–1^ (National Oceanic and Atmospheric Administration NOAA, 2019). It would possibly reach up to 936 μmol mol^–1^ in 2100 under RCP8.5 scenario (IPCC, 2014). Rice (*Oryza sativa L.*) has been one of the most important crops for human beings since the 1900s, especially in Asia and in the tropical and subtropical regions of Africa. Rice has the third largest planting area in the world and has been the main source of daily food for nearly half of the world’s population. With Global (CO_2_) rising and the increase of global population, it is critical to investigate and predict the responsive changes of the physiology and growth of rice to the increasing (CO_2_).

As the essential substrate for photosynthesis, the increase of (CO_2_) will stimulate photosynthesis and increase the biomass production and yield (Ainsworth and Long, 2005; Ainsworth and Rogers, 2007; Brito et al., 2020). Soil-Plant-Atmosphere-Research (SPAR) showed that rising (CO_2_) from 350 to 700 μmol mol^–1^ increased rice growth, grain yield and canopy photosynthesis and increased the final aboveground biomass by 29% with sufficient irrigation (Baker and Allen, 2005). The increase of air temperature at future elevated (CO_2_) made the CO_2_ effect on photosynthesis more complicated. Leaf photosynthetic rate (A) of two species, rice and soybean, were both increased under (CO_2_) enrichment, but this enhancement was reduced when the air temperature increased by 3°C (Vu et al., 1997). Rising (CO_2_) from 370 to 700 μmol mol^–1^ could offset the negative effect on photosynthesis due to 3.0–3.9°C warming, but had larger negative effect on photosynthetic carboxylation capacity in warming condition compared with ambient air temperature (Lamba et al., 2018). The combination of rising (CO_2_) and temperature increased the photosynthesis but decreased yield for rice (Cai et al., 2016). The yield reduction under the combination of 200 μmol mol^–1^ above ambient (CO_2_) and 1°C warming was reported in another research and the decrease of spikelet density might be the dominant factor (Wang W. et al., 2018).

Stomatal function is an important factor affecting photosynthesis and transpiration of plants. Lower stomatal conductance (g_s_) and transpiration rate (TR), therefore higher water use efficiency (WUE) due to elevated (CO_2_) were reported in varieties of species (Wand et al., 1999; Medlyn et al., 2001; Ainsworth and Rogers, 2007; Shimono and Bunce, 2009). Free air concentration enrichment (FACE) experiments showed an average increase of 23% in yield and a 25% reduction in g_s_ when (CO_2_) increased from 360 to 627 μmol mol^–1^ (Ainsworth, 2008). FACE experiments in Iwate, Japan, indicated that raising (CO_2_) by 200 μmol mol^–1^ significantly decreased g_s_ by 23% on average and doubling (CO_2_) made transpiration of rice reduced by 45 and 41% at full heading stage and mid-ripening stage, respectively (Shimono and Bunce, 2009; Shimono et al., 2010). Another research showed that lower g_s_ of wheat grown under ambient (CO_2_) was observed compared with that under elevated (CO_2_) when ammonium fertilization was supplied as the sole N source, and this phenomenon disappeared when plants were set under nitrate nutrition (Torralbo et al., 2019). And EucFACE in 2017 found no significant influence on g_s_ for one dominant C_3_ grass and two sympatric C_3_ forbs due to increasing (CO_2_) by 150 μmol mol^–1^ (Pathare et al., 2017). Therefore, the regulation of stomatal conductance by elevated CO_2_ could vary greatly depending on other environmental factors.

Responses of plant photosynthesis to short-term changes in environmental factors can be reflected through diurnal variations in photosynthesis. The maximum of A_net_ could be observed at 9 a.m. in tea [*Camellia sinensis* (*L*.) *O. Kuntze*] (Mohotti and Lawlor, 2002). An Conviron environmental chamber experiment for drought-tolerant plant *Elaeagnus umbellata* showed that A_net_ and g_s_ both reached the maximum rates early in the day (around 10 a.m.) and the daily average levels decreased significantly during times of drought stress (Naumann et al., 2010). Research for maize showed that the maximum of A_net_ and g_s_ occurred from 12 p.m. to 15 p.m. among two N fertilized and non-fertilized treatments, and the difference of A_net_ was significant due to N treatment when the light intensity was high, while the influence was found minimal on g_s_ and C_*i*_ (Peng et al., 2014). FACE research focused on maize found no significant difference in the diurnal variation of A_net_ at ambient and elevated (CO_2_) in the early growing stage, but the difference could be observed in the late growing stage (Markelz et al., 2011). Depression of net CO_2_ assimilation occurred around noon due to stomatal closure and the decrease of intercellular CO_2_ (C_*i*_) and elevated (CO_2_) could either significantly enhance the assimilation ability or weaken the midday depression (Nijs et al., 1992; Špunda et al., 2005). However, when C_3_ plants were exposed to elevated (CO_2_) for an extended period of time, the accelerated rate of photosynthesis often cannot be maintained, a phenomenon called photosynthetic acclimation to elevated (CO_2_) (Van Oosten and Besford, 1996). Ignoring this acclimation would possibly overestimate the magnitude of photosynthetic stimulation of elevated (CO_2_) concentrations compared with what was expected with the influence of short-term elevated (CO_2_) (Dusenge et al., 2019). Photosynthetic acclimation was usually associated with the down-regulation of stomatal conductance, Rubisco amount or activity, nitrogen concentrations or sink strength under elevated CO_2_ conditions (Ainsworth et al., 2004; Leakey et al., 2009; Rogers et al., 2017). For plants holding nitrogen-fixing bacteria, reduction of photosynthesis will be effectively alleviated under long-term elevated (CO_2_), especially under nitrogen limitation (Rho et al., 2020). Few studies have investigated the diurnal variations of photosynthetic characteristics of rice throughout the growing season. Whether the effects varied across different cultivars and growing stages requires systematic measurements of the diurnal variation of photosynthesis and stomatal conductance throughout the growing season to shed lights on both the short- and long- term effect of CO_2_ on photosynthetic characteristics.

Understanding and predicting large-scale carbon, water, and energy cycles requires accurate simulations in leaf photosynthesis and stomatal activities (Laisk et al., 2005). The Farquhar-von Caemmerer–Berry model (FvCB model), Ball, Woodrow and Berry (BWB model), and Medlyn model (MED model) are widely used photosynthetic and stomatal models to simulate the photosynthesis and stomatal conductance at the leaf level (Farquhar et al., 1980; Ball et al., 1987; Medlyn et al., 2012). The parameters of these models (V_cmax_, J_max_, and R_d_ from the FvCB model, m and g_0_ from BWB model, g_1_ and g_0_ from MED model) are valuable for large-scale simulations and represent important physiological traits that determine plant photosynthetic potential and water-use efficiency. How do photosynthetic parameters of V_cmax_, J_max_, and R_d_ and stomatal slope parameters vary among different rice cultivars and under different CO_2_ conditions require further study and analysis. Whether using cultivar-specific or environment-specific, instead of generic, model parameters can increase the accuracy of both photosynthesis and stomatal conductance simulations needs further investigation too.

In this study, diurnal changes of net photosynthetic rate (A_net_), stomatal conductance (g_s_), intercellular CO_2_ concentration (C_*i*_) and chlorophyll a fluorescence characteristics (photochemical efficiency of PSII, F_v_′/F_*m*_′; actual photochemical efficiency of PSII, ΦPSII; photochemical quenching, qP) were measured in two rice cultivars grown in the open-top-chambers at ambient (∼450 μmol mol^–1^) and elevated (∼650 μmol mol^–1^) CO_2_ concentrations throughout the growing season for 2 years. Seasonal variations of V_cmax_, J_max_, and R_d_ and stomatal slope parameters were determined by A_net_-C_*i*_ measurements at different growing stages. The objectives of this study were: (1) to investigate the diurnal and seasonal variations of rice photosynthetic performance under future atmospheric CO_2_ conditions; (2) to compare the diurnal and seasonal variations across two rice cultivars; (3) to test whether the photosynthetic and stomatal conductance models are effective under elevated CO_2_ conditions and whether using cultivar-, CO_2_- and stage- specific parameters can improve the accuracy of the photosynthesis and stomatal conductance simulations. Specifically, we hypothesized that (1) the positive effects of elevated (CO_2_) observed in the earlier growing season will decrease in the later growing season; (2) the extent of photosynthetic acclimation will be smaller for the more CO_2_-responsive cultivar; (3) the cultivar-, CO_2_-, and stage- specific parameters will increase the accuracy of the photosynthetic and stomatal conductance models.

## Materials and Methods

### Site Description

The study site was located in the agrometeorological experimental station of Nanjing University of Information Science and Technology, in Nanjing, Jiangsu province of China (32°16′N, 118°86′E). The climate in this region characterizes subtropical monsoon season, with the annual average precipitation of 1,100 mm, the average temperature in recent years of 15.6°C and the average annual frost-free period of 237 days. The soil texture in the tillage layer was loamy clay and the clayey content was 26.1%. The bulk density of 0–20 cm soil was 1.57 g⋅cm^–3^ and the pH (H_2_O) value were 6.3. The organic carbon and total nitrogen content were 11.95 and 1.19 g⋅kg^–1^, respectively.

### Experimental Design

Open top chambers (OTC) were used in the experiment to simulate elevated (CO_2_) treatments and description was provided in Supplementary Material 1. The OTCs are regular octagonal prisms with a diameter of 3.75 m, a height of 3 m, and a base area of 10 m^2^. There were two (CO_2_) treatments, ambient [a(CO_2_)] and elevated [e(CO_2_)], each with four replicative chambers. The treatment of elevated (CO_2_) started from the turning green stage and lasted to the end of growing season. The (CO_2_) concentration in the OTCs was controlled with an automatic control platform, composed of CO_2_ sensors, gas-supplying devices and automatic control system. Three wind-blowing fans were placed in each chamber to make the CO_2_ gas in the chamber evenly distributed and the top of the OTC is designed with an opening inclined 45° inward to avoid the rapid loss of CO_2_ gas. The CO_2_ sensor fed back the surrounding CO_2_ concentration information in the chambers to the automatic control system every 2 s. Our experiment was performed from 2019 to 2020 to make sure that the trend we observed was not a random impact. The daytime (CO_2_) averaged over the growing season was 641 ± 43 and 631 ± 39 μmol mol^–1^ in elevated (CO_2_) chambers and 478 ± 34 and 485 ± 33 μmol mol^–1^ in ambient (CO_2_) chambers in 2019 and 2020, respectively. The nighttime (CO_2_) was 667 ± 27 and 673 ± 24 μmol mol^–1^ in elevated (CO_2_) chambers and 537 ± 49 and 550 ± 36 μmol mol^–1^ in ambient (CO_2_) chambers.

Two rice cultivars, Yangdao6, a more CO_2_-responsive indica cultivar and Wuyunjing30, a less CO_2_-responsive japonica cultivar, as indicated in previous studies (Zhu et al., 2015; Tao and Wang, 2021; Wang et al., 2021), were selected in this study. Seedlings of each rice cultivar were transplanted into the eight OTCs on June 19, 2019 and June 16, 2020, and harvested on October 25, 2019 and October 28, 2020, respectively. The spacing of the hills was 16.7 cm × 25 cm (equivalent to 24 hills m^–2^). During the whole growing season, sufficient supplies of water and fertilizer were maintained.

### Photosynthetic Measurements

Gas exchange characteristics (including net photosynthetic rate and stomatal conductance) were measured with a portable infrared gas analyzer (LI-COR 6400LCF; LI-COR, Lincoln, NE, United States) on one randomly selected and fully expanded healthy leaf of each cultivar from each chamber. The measurement was taken at jointing, booting, heading, grain-filling and maturity stage on July 24, August 18, September 8, September 28 and October 13 in 2019, and July 25, August 24, September 7, September 24 and October 20 in 2020, respectively, which were confirmed by observation and previous research (Counce et al., 2000; IRRI, 2013). During diurnal measurements, the (CO_2_) of the leaf chambers were set according to the OTC conditions and the temperature and light levels were set as the environmental conditions. Photochemical efficiency of PSII in light-adapted leaves (F_v_′/F_*m*_′) and photochemical quenching (qP) were measured using a Licor 6400-40 Leaf Chamber Fluorometer. The photosynthesis-CO_2_ response (A_net_–*C*_*i*_) curves were measured on the next day after the diurnal measurements were taken. During measurements, leaves were acclimated for 30–60 min before adjusting the CO_2_ concentrations. Thereafter, CO_2_ concentration was decreased in six steps (400, 300, 230, 150, 90, and 50 μmol mol^–1^ CO_2_) and then increased in four steps (400, 600, 800, and 1,000 μmol mol^–1^ CO_2_). Leaf temperature was controlled at 35, 35, 35, 30, and 25°C when A_net_–*C*_*i*_ curve measurement was conducted at jointing, booting, heading, grain-filling, and maturity stage, respectively, and photosynthetic photon flux density (PPFD) was maintained at 2,000 μmol m^–2^ s^–1^ all the time because the light level is close to the midday light level in the region.

### Assessing the Photosynthetic and Stomatal Conductance Parameters

A_net_-C_*i*_ curves were fit to the FvCB models (Equations 1, 2) to solve the photosynthetic parameters including maximum ribulose 1⋅5-bisphosphate carboxylase/oxygenase (Rubisco) carboxylation rate (V_cmax_, μmol m^–2^ s^–1^), potential light saturated electron transport rate (J_max_, μmol m^–2^ s^–1^), and leaf dark respiration (R_d_, μmol m^–2^ s^–1^), respectively. A_net_-C_*i*_ curves were then fit to the BWB (Equation 3) and Medlyn (Equation 4) models to solve the parameters of m and g_1_ (Wolz et al., 2017; Wang D. et al., 2018).

(1) $$A_{\text{net}}=\left(1-\frac{\Gamma^{*}}{C_{i}}\right)\,\frac{V_{\text{cmax}}\cdot C_{i}}{C_{i}+K_{C}\,\left(1+O/K_{O}\right)}-R_{d}$$

where C_*i*_ is the intercellular (CO_2_), O is the oxygen concentration, Γ^∗^ is the photosynthetic CO_2_ compensation point without dark respiration, K_*C*_ and K_*O*_ are the Michaelis–Menten constants for CO_2_ and O_2_ and can be found in Bernacchi et al. (2001). Equation 1 can be used if A_net_ is limited by Rubisco activity [low (CO_2_)] and if A_net_ is limited by the regeneration of ribulose-1,5-bisphosphate (RuBP) at high (CO_2_), it can be determined as follows:

(2) $$A_{\text{net}}=\left(1-\frac{\Gamma^{*}}{C_{i}}\right)\,\frac{J\cdot C_{i}}{{\mathrm{aC}}_{i}+b\,\Gamma^{*}}-R_{d}$$

where a and b are model parameters and are given as 4.0 and 8.0, respectively (Bernacchi et al., 2003); J is the photosynthetic electron transport rate and can be used to estimate J_max_ with the non-rectangular hyperbolic model (von Caemmerer, 2000). Values of both V_cmax_ and J_max_ would be corrected to those at 25°C after the estimation by using Arrhenius equation provided from Bernacchi et al. (2003).

For each leaf, a linear least squares regression of Equations 3 or 4 was used to estimate the intercept and slope parameters of the BWB and MED model, respectively. Biologically, the slope parameter of each model represents the sensitivity of *g*_s_ to changes in A_net_, C_*a*_, and atmospheric water status and will be the focus of this analysis. A term for the *y* intercept of each model algorithm (*g*_0_) can be used to describe variation in minimum *g*_s_. Only simulations that provided a regression between modeled and observed stomatal conductance with an *R*^2^ > 0.8 were included in further analyses (Wolz et al., 2017).

(3) $$g_{s}=g_{0}+m\,\frac{\mathrm{Ah}}{C_{a}}$$

where *g*_s_ is stomatal conductance (mol m^–2^ s^–1^), *A* is the net rate of photosynthetic CO_2_ uptake (μmol m^–2^ s^–1^), *h* is atmospheric relative humidity (unitless), *C*_*a*_ is the atmospheric CO_2_ concentration at the leaf surface (μmol mol^–1^), *g*_0_ is the y-axis intercept and *m* is the slope of the line.

(4) $$g_{s}=g_{0}+1.6\,\left(1+\frac{g_{1}}{\sqrt{D}}\right)\,\frac{A}{C_{a}}$$

where D is the atmospheric vapor pressure deficit (kPa) and g_1_ is the model parameter related to the slope of the line. Previous study showed that whether the value of g_0_ is 0 does not significantly affect the linear and non-linear relationship in Equations 3, 4 (Leakey et al., 2006), so we set the value of g_0_ to 0 in order to focus on the variations of the stomatal slope.

The diurnal measurements of A_net_ and g_s_ were used to validate the coupled FvCB photosynthetic model and BWB and MED stomatal conductance models (Wolz et al., 2017). Specific (cultivar-, CO_2_- and stage- specific) photosynthetic and stomatal parameters and generic parameters (the average values across all the groups) were used to test whether the specific parameters will increase the accuracy of the models.

### Statistical Analysis

Four-way analysis of variance (ANOVA) was used to test the fixed effects of years, cultivars, growing stages, CO_2_ treatments and their interactions on A_net_, g_s_, F_v_′/F_m_′, V_cmax_, J_max_, R_d_ and stomatal slope parameters. *Post hoc* Tukey’s HSD tests were conducted on specific contrasts to examine significant treatment effects among groups. General linear models (GLM) were used to assess the relationship between observed and modeled parameters. For all the analysis, the normality of the residuals was tested using the Shapiro–Wilk test. All statistical testes were considered significant at *P* ≤ 0.05. Mean values of each variable were expressed with their standard error (SE). All analyses were conducted in R with package “plantecophys” and all figures were drawn with the “ggplot” function in package “tidyverse” (R 3.6.3^1^).

## Results

### Diurnal and Seasonal Variations in Environmental Parameters

**FIGURE 1:**
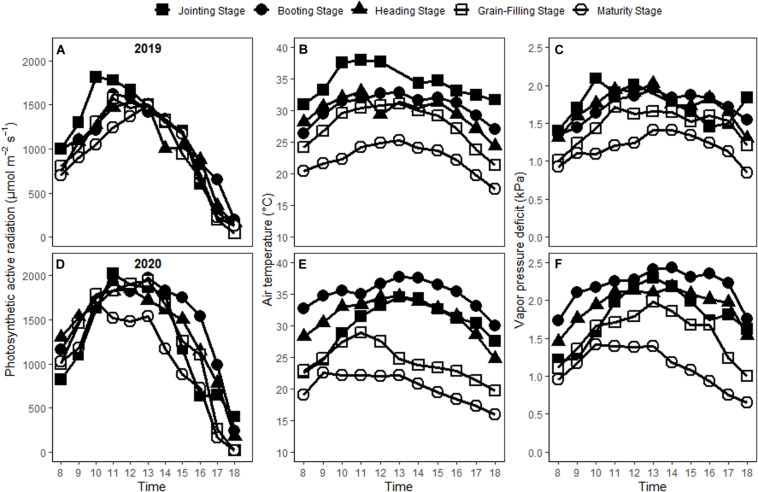
Changes of photosynthetic active radiation **(A,D)**, air temperature **(B,E),** and vapor pressure deficit **(C,F)** with time on the measuring date at five different growing stages in 2019 and 2020.

The photosynthetic active radiation (PAR) usually reached its maximum from 10 a.m. to 13 p.m. (Figure 1). The maximum values of PAR varied on the measuring date across the growing season, with 1,809.96, 1,619.51, 1,519.59, 1,574.90, and 1,489.53 μmol m^–2^ s^–1^ at jointing, booting, heading, grain-filling and maturity stages in 2019 and 2,019.40, 1,919.95, 1,850.80, 1,940.95, and 1,701.12 μmol m^–2^ s^–1^ in 2020, respectively. Diurnal variations of air temperature (T_air_) and vapor pressure deficit (VPD) followed the similar trend as PAR. The maximal T_air_ were 38.03, 32.91, 32.97, 31.06, and 25.28°C in 2019 and 34.50, 37.71, 34.62, 28.92, and 22.57°C in 2020, while those of VPD were 2.09, 1.96, 2.02, 1.72, and 1.41 kPa in 2019 and 2.29, 2.42, 2.18, 1.98, and 1.42 kPa in 2020 on the measuring date at five growing stages. T_air_, VPD and PAR varied significantly across the growing season.

### Diurnal and Seasonal Variations in A_net_ and g_s_

**FIGURE 2:**
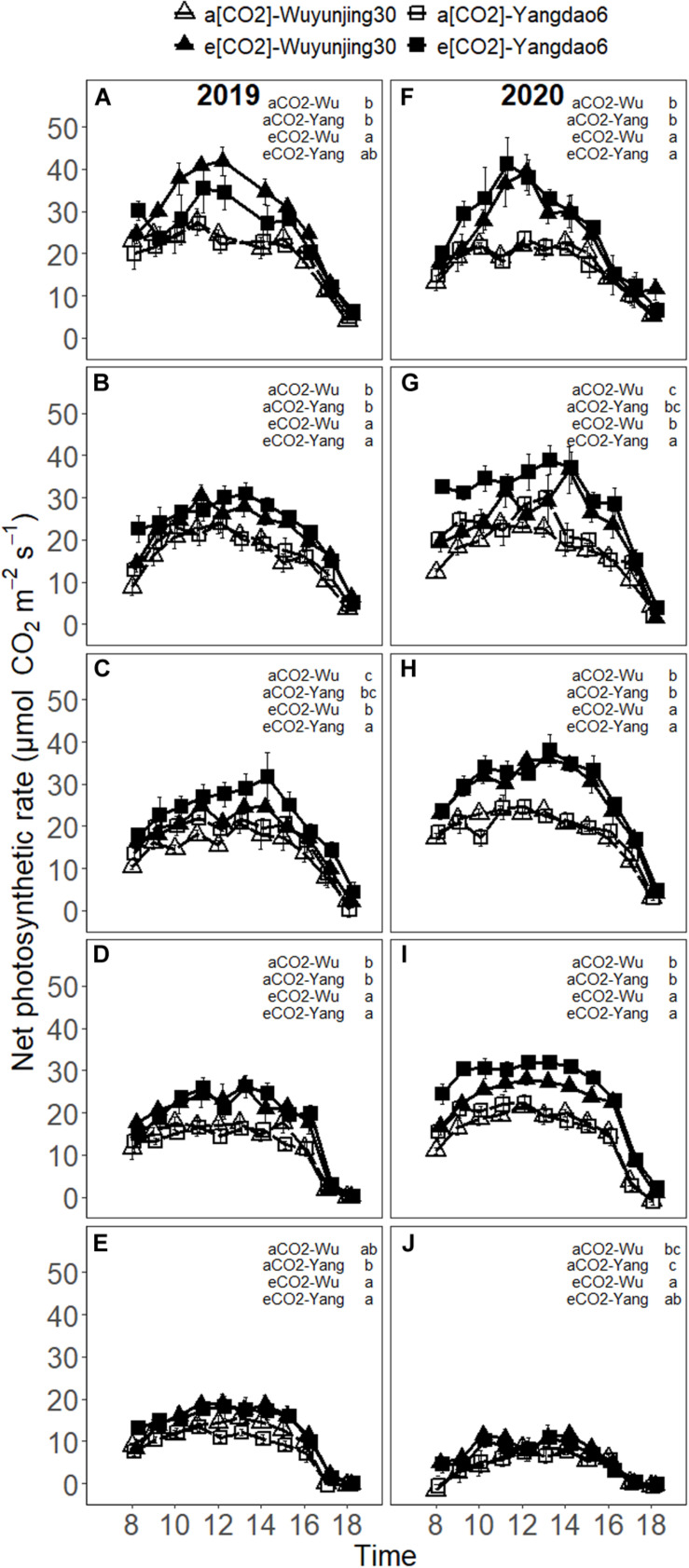
Diurnal variations in net photosynthetic rate (A_net_) of rice cultivar Wuyunjing30 and Yangdao6 grown at ambient and elevated (CO_2_) at jointing **(A,F)**, booting **(B,G)**, heading **(C,H)**, grain-filling **(D,I),** and maturity **(E,J)** stages in 2019 and 2020. Values were expressed as means ± standard errors form. Values of A_net_ under four (CO_2_) × cultivar treatments at 13 p.m. at jointing stage in 2019 were deleted because of the abnormal values found by examination. Statistical analyses of multiple comparisons for five growing stages in 2 years were shown in each panels of the figure in the form of lowercase letters.

In most cases, A_net_ increased to the maximum around noon and decreased in the afternoon and the maximum value appeared no later than 14 p.m. (Figure 2). The midday depression of A_net_ was observed on some of the measuring dates for both the cultivars and (CO_2_) treatments. On average, elevated (CO_2_) significantly stimulated A_net_ of both cultivars by 34.19% in 2019 and 47.65% in 2020 in the whole growing season, which was consistent with the impact of elevated (CO_2_) on intercellular (CO_2_) (Supplementary Material 2). The greatest stimulation of elevated (CO_2_) on A_net_ could be observed around midday at jointing stage (54.35% in 2019 and 56.12% in 2020 for Wuyunjing30; 57.95% in 2019 and 61.50% in 2020 for Yangdao6), and the enhancement of A_net_ of each cultivar due to elevated (CO_2_) at the following four growing stages were much lower than that at this stage in 2 years (Supplementary Material 3). A_net_ decreased significantly with the advance of growing season and there were significant (CO_2_) × growing stage effect on A_net_ (Table 1 and Supplementary Material 4). The more CO_2_-responsive cultivar Yangdao6 had higher A_net_ than Wuyunjin30 and the two cultivars responded similarly to elevated (CO_2_) (Supplementary Material 5).

**TABLE 1:** The analysis of variance (ANOVA) of photosynthetic characteristics and model parameters.

Parameter	A_net_	g_s_	F_v_′/F_m_′	qP	V_cmax_	J_max_	R_d_	m	g_1_
(CO_2_)	***	ns	***	*	ns	ns	ns	*	*
Cultivar	*	*	***	***	ns	ns	ns	**	**
Stage	***	***	***	***	**	***	**	***	ns
Year	*	*	***	***	ns	ns	ns	ns	ns
(CO_2_) × cultivar	ns	ns	ns	ns	ns	ns	ns	ns	ns
(CO_2_) × stage	**	ns	ns	ns	ns	ns	ns	ns	ns
Cultivar × stage	ns	***	ns	ns	ns	ns	ns	**	*
(CO_2_) × year	*	ns	ns	ns	ns	ns	ns	ns	ns
Cultivar × year	ns	+	*	ns	ns	ns	ns	ns	ns
Stage × year	**	ns	ns	ns	ns	ns	ns	ns	ns

***Pr(>F) < 0.001; **Pr(>F) < 0.01; *Pr(>F) < 0.05; +Pr(>F) < 0.1; ns, not significant.

**FIGURE 3:**
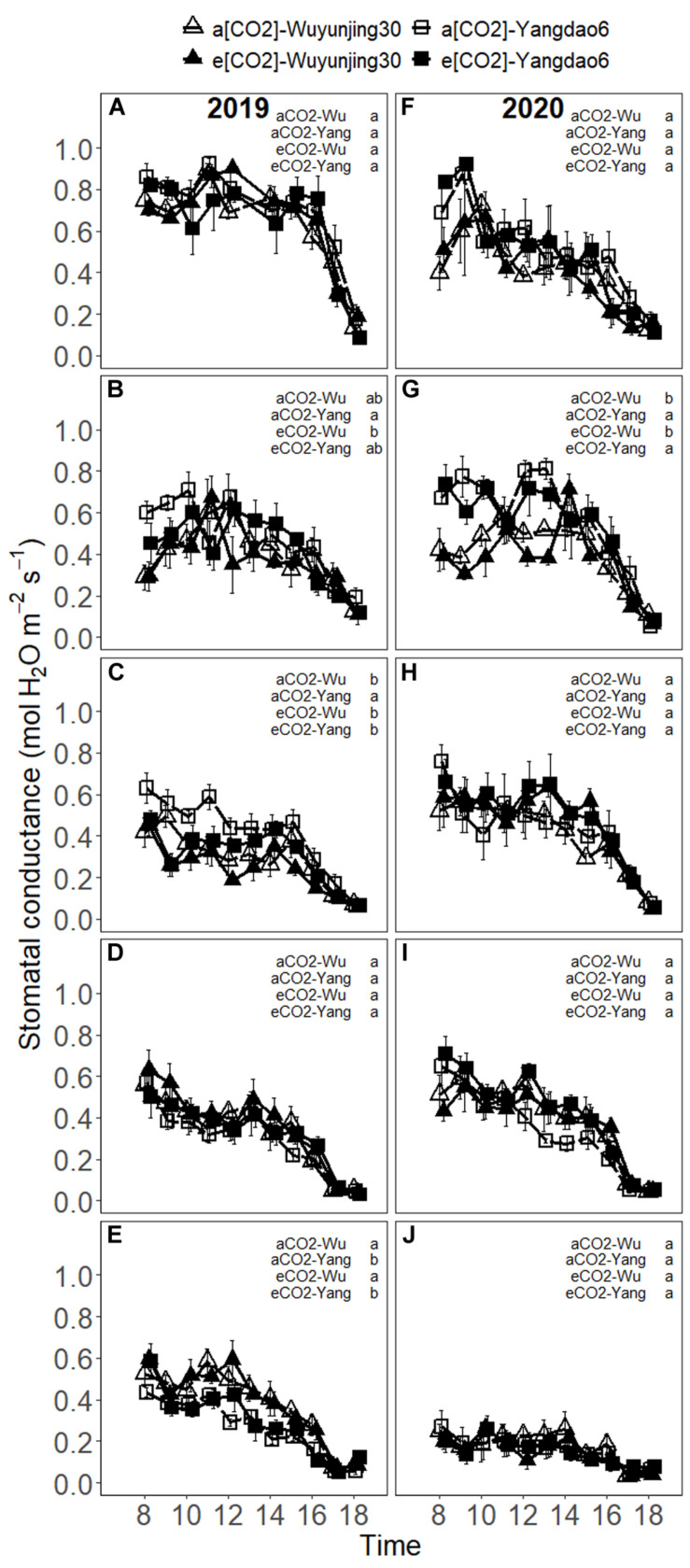
Diurnal variations in stomatal conductance (g_s_) of rice cultivar Wuyunjing30 and Yangdao6 grown at ambient and elevated (CO_2_) at jointing **(A,F),** booting **(B,G)**, heading **(C,H)**, grain-filling **(D,I),** and maturity **(E,J)** stages in 2019 and 2020. Values were expressed as means ± standard errors form. Values of g_s_ under four (CO_2_) × cultivar treatments at 13 p.m. at jointing stage in 2019 were deleted because of the abnormal values found by examination. Statistical analyses of multiple comparisons for five growing stages in 2 years were shown in each panels of the figure in the form of lowercase letters.

The values of g_s_ remained relatively high before 15 p.m. and started to decrease after that (Figure 3). In 2019, g_s_ under all (CO_2_) × cultivars tended to decrease significantly with the advance of growing season, while a slight recovery of g_s_ beginning at heading stage was observed for Wuyunjing30 under both (CO_2_) treatments (Supplementary Material 4). In 2020, g_s_ of Yangdao6 under both (CO_2_) treatments slightly increased from jointing to booting stage, while that of Wuyunjing30 decreased or remained stable during this period, and g_s_ always decreased from heading to maturity stage. Seasonal variations in g_s_ synchronized well with those in A_net_, especially in 2020. Elevated (CO_2_) had no significant effect on g_s_ (Table 1 and Supplementary Material 3). The values of g_s_ of cultivar Yangdao6 were higher than those of Wuyunjing30 (Supplementary Material 5).

### Diurnal and Seasonal Variations in F_v_′/F_m_′, ΦPSII, and qP

**FIGURE 4:**
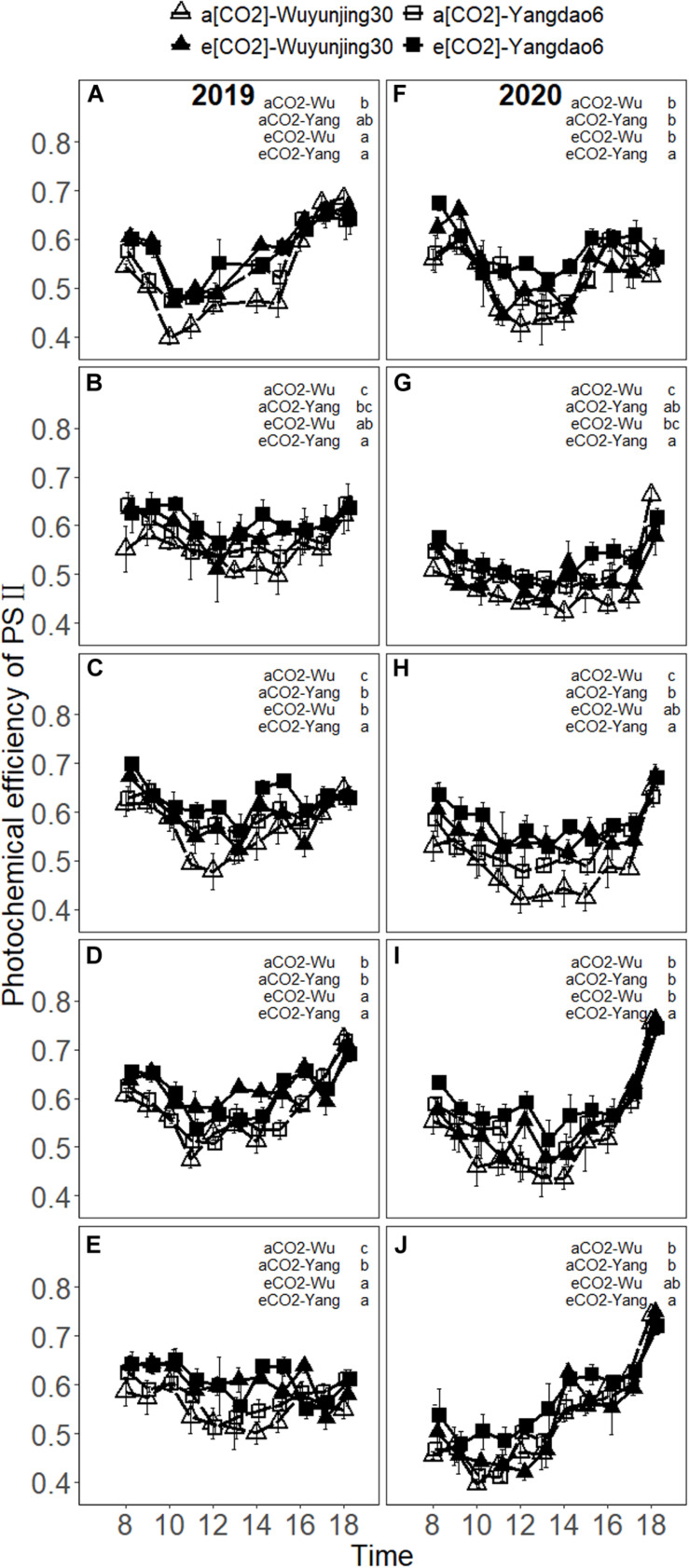
Diurnal variations in photochemical efficiency of PSII (F_v_′/F_m_′) of rice cultivar Wuyunjing30 and Yangdao6 grown at ambient and elevated (CO_2_) at jointing **(A,F)**, booting **(B,G)**, heading **(C,H)**, grain-filling **(D,I),** and maturity **(E,J)** stages in 2019 and 2020. Values were expressed as means ± standard errors form. Values of F_v_′/F_m_′ under four (CO_2_) × cultivar treatments at 13 p.m. at jointing stage in 2019 were deleted because of the abnormal values found by examination. Statistical analyses of multiple comparisons for five growing stages in 2 years were shown in each panels of the figure in the form of lowercase letters.

The values of F_v_′/F_m_′, ΦPSII, and qP decreased to the minimum around noon and recovered to normal or even higher levels under each treatment (Figure 4 and Supplementary Materials 6, 7). F_v_′/F_m_′ increased with the advance of growing season and reached the maximum at heading stage or grain-filling stage, and decreased at maturity stage in 2019 (Supplementary Material 4). In 2020, F_v_′/F_m_′ decreased to the minimum value at booting stage at first, then increased to the maximum value at heading or grain-filling stage, and decreased till the end of the growth period. On average, elevated (CO_2_) significantly increased F_v_′/F_m_′ of both cultivars by 6.92% in 2019 and 6.41% in 2020 across the growth season, while qP was decreased by 4.30% in 2019 and 3.97% in 2020, respectively (Table 1). The greatest stimulation influences on F_v_′/F_m_′ due to elevated (CO_2_) at midday were 21.39% at heading stage in 2019 and 26.80% at the same stage in 2020 for Wuyunjing30, and 16.18% at maturity stage in 2019 and 29.27% at grain-filling stage in 2020 for Yangdao6 (Supplementary Material 3). The values of F_v_′/F_m_′ of cultivar Yangdao6 were significantly higher than those of Wuyunjing30 (Supplementary Material 5).

### Seasonal Variations of Photosynthetic and Stomatal Parameters

For most treatments, V_cmax_ and J_max_ reached the maximum at heading stage and decreased to the minimum at maturity stage (Table 2), which could also be observed in A_net_-C_*i*_ curves (Supplementary Material 8). Values of V_cmax_ at booting stage were higher than those at jointing stage only for Yangdao6 in 2019. Values of J_max_ at booting stage were lower than those at jointing stage for Wuyunjing30 grown at ambient and elevated (CO_2_) and Yangdao6 grown at elevated (CO_2_) in 2019. The values of R_d_ increased to the maximum at grain-filling or maturity stage. There is no significant influence of (CO_2_) or cultivar or the interaction on V_cmax_, J_max_, and R_d_ (Table 2), and A_net_-C_*i*_ curves showed no differences of three parameters among four (CO_2_) × cultivar treatments, either (Supplementary Material 9). The values of m and g_1_ were highest at maturity stage across (CO_2_) × cultivar treatments. On average, elevated (CO_2_) significantly decreased m and g_1_ of both cultivars by 7.62 and 9.82% respectively. The values of m and g_1_ of more CO_2_-responsive cultivar Yangdao6 was 10.75 and 10.87% lower than those of less CO_2_-responsive cultivar Wuyunjing30, respectively.

**TABLE 2:** Model parameters determined from A_net_-Ci data in 2019 and 2020.

		2019				2020			
	Stage	a(CO_2_)		e(CO_2_)		a(CO_2_)		e(CO_2_)	
		Wuyunjing30	Yangdao6	Wuyunjing30	Yangdao6	Wuyunjing30	Yangdao6	Wuyunjing30	Yangdao6
V_cmax_	Jointing	73.4 ± 6.5	61.9 ± 6.9	68.3 ± 7.9	50.5 ± 2.9	77.2 ± 4.8	79.0 ± 5.6	73.5 ± 3.5	69.8 ± 5.1
	Booting	71.6 ± 4.0	66.6 ± 3.8	60.1 ± 5.1	70.7 ± 1.6	68.0 ± 0.4	71.3 ± 0.2	65.0 ± 7.2	61.5 ± 2.8
	Heading	70.8 ± 1.9	88.1 ± 4.5	79.4 ± 5.6	89.7 ± 4.7	87.3 ± 1.0	87.8 ± 2.6	76.7 ± 1.4	102.0 ± 1.8
	Grain-filling	75.0 ± 3.4	83.9 ± 5.0	78.6 ± 3.2	67.3 ± 4.4	71.7 ± 2.5	85.1 ± 2.0	68.5 ± 1.7	96.4 ± 17.7
	Maturity	29.5 ± 1.7	51.8 ± 2.5	40.3 ± 0.6	61.4 ± 2.1	46.2 ± 4.3	34.1 ± 1.1	56.1 ± 19.3	38.6 ± 4.6
J_max_	Jointing	212.7 ± 8.9	169.3 ± 10.0	206.5 ± 35.8	172.9 ± 12.6	173.4 ± 7.9	150.9 ± 2.6	182.5 ± 13.6	179.3 ± 19.0
	Booting	189.1 ± 10.6	204.4 ± 7.6	168.6 ± 9.8	169.4 ± 6.3	225.8 ± 8.7	225.5 ± 10.2	183.9 ± 19.5	195.8 ± 8.2
	Heading	180.3 ± 5.5	275.8 ± 24.8	203.4 ± 23.4	227.0 ± 13.7	247.6 ± 12.8	209.5 ± 13.5	193.1 ± 16.2	263.9 ± 20.2
	Grain-filling	174.2 ± 7.4	186.9 ± 15.7	190.3 ± 7.0	156.3 ± 10.6	164.0 ± 7.6	183.6 ± 6.4	144.5 ± 6.8	199.4 ± 49.2
	Maturity	76.1 ± 4.0	119.1 ± 6.6	96.5 ± 2.3	138.6 ± 3.7	97.8 ± 6.8	76.1 ± 1.6	63.0 ± 11.8	80.1 ± 10.5
R_d_	Jointing	1.8 ± 0.9	2.2 ± 0.9	0.1 ± 0.2	0.8 ± 0.4	4.8 ± 0.3	3.6 ± 0.4	4.5 ± 0.7	5.6 ± 1.1
	Booting	3.6 ± 1.3	0.4 ± 0.4	1.2 ± 0.4	0.5 ± 0.1	0.7 ± 0.6	0.4 ± 0.5	0.5 ± 0.5	0.2 ± 0.4
	Heading	2.0 ± 0.2	0.4 ± 1.2	5.4 ± 1.9	0.9 ± 0.4	0.6 ± 0.7	1.4 ± 0.8	0.2 ± 0.5	0.8 ± 0.6
	Grain-filling	3.2 ± 0.4	1.6 ± 0.4	3.5 ± 0.4	1.9 ± 0.5	4.6 ± 1.2	7.4 ± 2.7	4.0 ± 0.7	5.8 ± 1.7
	Maturity	3.8 ± 0.6	4.2 ± 0.5	4.0 ± 0.4	3.3 ± 0.4	6.1 ± 1.6	2.8 ± 0.3	4.2 ± 1.2	3.3 ± 0.8
m	Jointing	13.2 ± 1.4	13.0 ± 0.6	11.8 ± 0.5	13.2 ± 1.9	12.9 ± 0.8	10.7 ± 0.8	12.4 ± 1.1	11.3 ± 1.0
	Booting	13.7 ± 0.7	12.5 ± 1.9	12.8 ± 0.5	11.6 ± 2.4	12.6 ± 1.3	13.8 ± 1.3	13.1 ± 0.5	10.9 ± 0.2
	Heading	12.6 ± 1.8	12.8 ± 1.2	10.2 ± 0.8	12.9 ± 1.1	11.9 ± 0.9	12.2 ± 1.6	10.7 ± 1.0	10.9 ± 1.3
	Grain-filling	11.3 ± 1.1	10.0 ± 0.9	12.1 ± 1.0	10.8 ± 0.8	12.2 ± 1.1	13.3 ± 0.8	13.7 ± 2.2	10.9 ± 0.1
	Maturity	24.4 ± 2.1	12.4 ± 2.2	16.0 ± 1.1	10.9 ± 0.9	17.3 ± 3.0	16.1 ± 1.7	20.3 ± 0.7	14.9 ± 0.9
g_1_	Jointing	6.0 ± 0.8	5.9 ± 0.2	5.5 ± 0.2	6.1 ± 0.9	5.8 ± 0.3	4.5 ± 0.5	5.4 ± 0.4	5.2 ± 0.2
	Booting	6.5 ± 0.3	5.8 ± 1.1	5.9 ± 0.4	5.0 ± 1.3	6.2 ± 0.6	6.9 ± 0.8	6.5 ± 0.2	5.0 ± 0.2
	Heading	5.0 ± 0.8	5.7 ± 0.5	4.4 ± 0.4	5.1 ± 0.6	5.2 ± 0.4	5.4 ± 0.7	4.4 ± 0.4	4.4 ± 0.7
	Grain-filling	4.8 ± 0.7	3.9 ± 0.3	5.0 ± 0.5	4.1 ± 0.4	5.3 ± 0.5	5.2 ± 0.7	5.7 ± 1.0	4.1 ± 0.2
	Maturity	9.3 ± 0.7	4.7 ± 0.8	5.6 ± 0.3	4.2 ± 0.4	6.7 ± 1.1	6.5 ± 0.6	7.4 ± 0.5	5.5 ± 0.2

### The Photosynthesis and Stomatal Conductance Simulations

**FIGURE 5:**
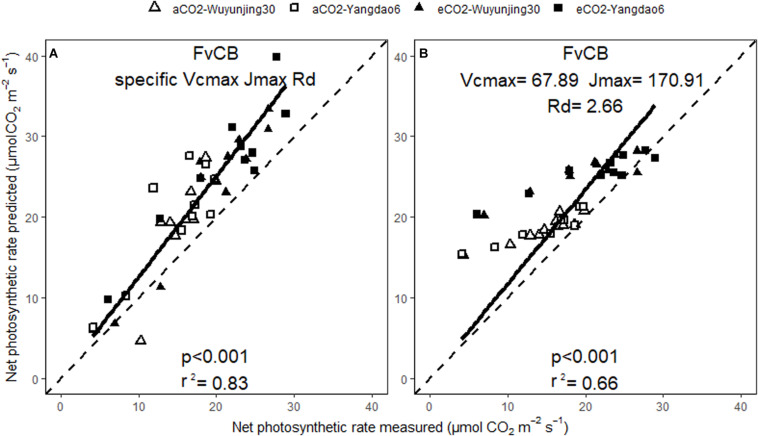
Relationships between the measured and simulated daily average net photosynthetic rate for the two rice cultivars at ambient and elevated (CO_2_) throughout the growing season. Net photosynthetic rate was simulated with FvCB model using either specific model parameters **(A)** or generic model parameters **(B)**. p value of the correlation analysis and the square of Pearson correlation coefficient (r^2^) are given.

**FIGURE 6:**
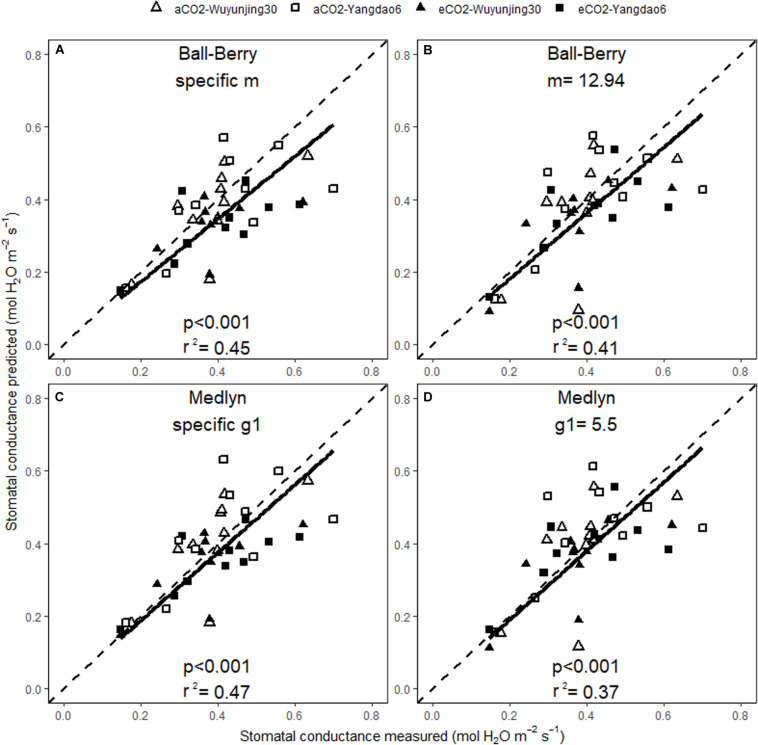
Relationships between the measured and simulated daily average stomatal conductance for the two rice cultivars at ambient and elevated (CO_2_) throughout the growing season. Stomatal conductance was simulated with BWB **(A,B)** or MED models **(C,D)**, using either specific model parameters **(A,C)** or generic model parameters **(B,D)**. p value of the correlation analysis and the square of Pearson correlation coefficient (r^2^) are given.

The predicted values of A_net_ and g_s_ correlated well with the measured ones across all treatments no matter whether BWB or MED was used or specific or generic model parameters were selected (Figures 5, 6). The square of Pearson correlation coefficient (r^2^) between predicted and measured values was used to describe the simulation accuracy in our study. We found that the accuracy of photosynthetic simulations was higher when using the specific parameters (*r*^2^ = 0.83) than using the generic ones (*r*^2^ = 0.66). The moderate improvement of g_s_ simulations was achieved using specific parameters (*r*^2^ = 0.45 for BWB and 0.47 for MED model), compared with using generic parameters (*r*^2^ = 0.41 for BWB and 0.37 for MED models).

## Discussion

Individual studies and meta-analysis have investigated the general tendency of elevated CO_2_ effects on plant physiology and production (Ainsworth and Long, 2005; Ainsworth, 2008; Wang et al., 2012). However, it remains unclear how the effects of elevated CO_2_ varied under short- and long- term treatment and across different rice cultivars. In this study, we took the diurnal and A_net_-C_*i*_ photosynthetic measurements throughout the growing season for 2 years. Overall, we found that (1) the diurnal variation of net photosynthetic rate was mainly affected by the dynamic of photosynthetic active radiation and air temperature and the midday depression of net photosynthetic rate observed in most cases was caused by both stomatal and non-stomatal factors; (2) CO_2_ had a positive effect on net photosynthetic rate and the effects decreased after the booting stage and the degree of the acclimation did not vary between the two tested cultivars; (3) the photosynthetic and stomatal conductance models proved to be effective under elevated CO_2_ conditions and using specific parameters greatly improved the accuracy of the photosynthetic simulations and moderately improved that of stomatal conductance simulations.

In general, values of A_net_ were lower in the early morning and the late afternoon and the maximum occurred around noon. These unimodal or bimodal patterns were consistent with the dynamics of PAR and T_air_ across growing stages. In contrary, although the decrease of g_s_ was synchronous with that of A_net_ in the afternoon, the variation of g_s_ in the morning could not reflect the dynamic of A_net_ in this period. Therefore, light intensity as well as air temperature, rather than stomatal functions, were the dominant factors affecting the diurnal variation of A_net_ on daily scale (Wen et al., 1998). The midday depression of A_net_ was observed in most cases in these 2 years and the synchronous decline of g_s_ could also be found when midday depression of A_net_ occurred, which was consistent with previous studies (Farquhar and Sharkey, 1982; Panda, 2011; Koyama and Takemoto, 2014). Short-term changes of C_*i*_ should be considered when determining whether stomatal or non-stomatal limitation caused the midday depression (Farquhar and Sharkey, 1982). Stomatal limitation would be the major factor if the reduction of C_*i*_ and g_s_ was synchronously observed when the midday depression of A_net_ occurred. In contrary, if C_*i*_ kept at the stable value or even increased, non-stomatal limitation should be taken into account. In our research, for example, rapid decrease of C_*i*_ could be observed under the situation of photosynthetic midday depression for Wuyunjing30 under both ambient and elevated (CO_2_) at heading stage in 2019. But C_*i*_ changed little or remained unchanged, for Wuyunjing30 and Yangdao6 under ambient (CO_2_) at jointing stage in 2020. Meanwhile, we noticed the decrease of F_v_′/F_m_′ and qP around noon for each cultivar at both the (CO_2_) treatments and they recovered to normal or even higher values at late afternoon, proving the existence of reversible photoinhibition in rice throughout the growth season (Adams and Demmig-Adams, 1992; Demmig-Adams and Adams, 1992). Liu et al. (2020) demonstrated that the midday depression of A_net_ in leaves of *Drepanostachyum luodianense* was mainly caused by non-stomatal limitation in April, but caused by stomatal limitation in July. And in September, stomatal limitation was the dominant factor leading to the A_net_ depression from 10 a.m. to 12 p.m. while non-stomatal limitation dominated the depression from 12 p.m. to 13 p.m. Therefore, the midday depression of photosynthesis was caused by both the stomatal and non-stomatal limitations such as photoinhibition, and the dominant factor changed depending on the surrounding environment. While the dynamics of major environmental factors such as PAR and air temperature affected the overall trend of the diurnal variation of rice photosynthesis, stomatal closure and photoprotection of plants responding to high light intensity and other non-stomatal limitations were the dominant factors affecting the midday decrease of the photosynthesis.

The stimulation of elevated (CO_2_) on plant photosynthesis has been widely reported in recent decades, but the magnitude of the stimulation varied greatly depending on species, plant functional types (PFTs) and other environmental factors (Ainsworth and Long, 2005; Wang et al., 2012). In this study, elevated (CO_2_) enhanced A_net_ of rice by 34.19 and 47.65% on average throughout the growing season in 2019 and 2020, respectively, which was comparable to what was reported 33% increase in the meta-analysis studies (Wand et al., 1999; Ainsworth and Long, 2005). Higher intercellular CO_2_ concentration (C_*i*_) and photochemical efficiency of PSII (F_v_′/F_m_′) were the main factors for the stimulation on A_net_ of rice at elevated (CO_2_). Photosynthetic acclimation to elevated (CO_2_) was found in crop varieties with limited sink size (Thilakarathne et al., 2015; Ruiz-Vera et al., 2017), but not in those with many large leaves (Ruiz-Vera et al., 2017) or high tillering potentials (Tausz-Posch et al., 2015). The enhancement of A_net_ due to elevated (CO_2_) was greatest at jointing stage in 2 years, but was weakened afterward and even disappeared at maturity stage in 2020, which suggested the occurrence of photosynthetic acclimation at elevated (CO_2_) and it could happen at the early growing season. We did not observe the less enhancement by elevated (CO_2_) on F_v_′/F_m_′ and qP at the following growing stages compared with jointing stage or any significant influence of elevated (CO_2_) on g_s_, V_cmax_ and J_max_ which could explain the early season photosynthetic acclimation. Therefore, the down-regulation of stomatal and Rubisco enzyme functions might not be the only reasons for the photosynthetic acclimation of rice at elevated (CO_2_) (Chen et al., 2005). A more frequent and detailed measurement in the early growing season was required to fully understand the evident but decreased elevated CO_2_ effect on the net photosynthesis. The elevated CO_2_ effect on rice roots growth, morphology and physiology was also under investigation in this study to further discover whether the photosynthetic acclimation of rice was the added-up effects from both the above- and below- ground physiological changes. Besides, there was no significant difference of the photosynthetic acclimation between more CO_2_-responsive cultivar Yangdao6 and less CO_2_-responsive cultivar Wuyunjing30 in terms of the occurrence and the extent of the acclimation, even though higher A_net_ and g_s_ were observed in Yangdao6 compared with Wuyunjing30 throughout the growing season, which was consistent with previous studies (Zhu et al., 2015).

Model parameters in FvCB model (V_cmax_, J_max_, and R_d_) and stomatal slope parameters in BWB model (m) and MED model (g_1_) varied across growing stages. The decrease of V_cmax_ and J_max_ could be the reason for the decline of photosynthetic capacity with the advance of growth period. V_cmax_, J_max_, and R_d_ varied not only among different species (Wullschleger, 1993), but also within single species in different environments (Bunce, 2000; Medlyn et al., 2002a; Medlyn et al., 2002b). Elevated (CO_2_) did not lead to significant changes of V_cmax_ and J_max_ in our research. The response of V_cmax_, J_max_, and R_d_ in C_3_ plants grown at elevated (CO_2_) were reported to be quite different and both stimulation and inhibition of elevated (CO_2_) effects had been reported in recent years (Sims et al., 1998a; Sims et al., 1998b; Ellsworth et al., 2004). Elevated (CO_2_) treatment as short as 7 days increased V_cmax_ and J_max_ for cucumber by 12.0 and 14.7% without drought stresses, but the enhancement was no longer significant under mild and severe drought stresses (Liu et al., 2018). From our ANOVA analysis, V_cmax_, J_max_, and R_d_ of rice did not vary significantly across cultivars, suggesting that photosynthetic simulations could use similar parameters at least between the two tested cultivars. For stomatal conductance models, previous research has reported significant correlation between stomatal slope parameters and environmental factors such as temperature, moisture and season and plant physiological factors such as wood density (Lin et al., 2015). And recent research has also indicated the variability of stomatal slopes in plants at species level (Wolz et al., 2017). FACE research reported no stomatal acclimation for soybean at elevated (CO_2_) (Leakey et al., 2009). However, the Ball–Berry slope was significantly different between ambient and elevated (CO_2_) grown wheat (Tausz-Posch et al., 2013). In our research, stomatal slopes (m and g_1_) varied across both (CO_2_) treatments and cultivars, demonstrating that variability of m and g_1_ was caused not only by the diversity of plant function groups but also by the diversity of physiological characteristics among cultivars within a single species.

Higher accuracy of simulation using specific model parameters in both photosynthesis model (V_cmax_, J_max_, and R_d_) and stomatal conductance model (m and g_1_) compared with using generic parameters was achieved in this study, suggesting that future simulations in large scale carbon and water cycles should take account of the variations of model parameters across environmental and non-environmental factors. Though we did not observe a significant impact of elevated (CO_2_) on V_cmax_ and J_max_, it should be noted that not considering the impact of mesophyll conductance (g_m_) might lead to the potential increased systematic errors of determining V_cmax_ and J_max_ (Singsaas et al., 2003). And the possibility that the differences of model parameters of rice will change under other environmental conditions should not be ruled out too. Therefore, further research focusing on the diversity of model parameters are needed to confirm the existence of (CO_2_), cultivar and growing stage variation of photosynthesis and stomatal conductance model parameters of rice and other plant species.

In conclusion, diurnal variations of net photosynthetic rate of rice showed unimodal or bimodal patterns, which was mainly influenced by light intensity and air temperature. Meanwhile, the closure of stomata and the photoinhibition around noon were dominant factors for the short-term midday depression of net photosynthetic rate. Elevated (CO_2_) greatly increased net photosynthetic rate at jointing stage. This stimulation was acclimated with the advance of growing season and was not affected by either stomatal limitation or Rubisco activity. Model parameters in photosynthesis model (V_cmax_, J_max_, and R_d_) and two stomatal conductance models (m and g_1_) varied across growing stages and m and g_1_ also varied across (CO_2_) treatments and cultivars, which led to more accurate photosynthesis and stomatal conductance simulations when using these specific parameters. The results in the study suggested that further researches are still needed to investigate the dominant factors contributing to the acclimation of photosynthetic capacity under future elevated CO_2_ conditions. The study also highlighted the need of investigating the impact of other environmental, such as nitrogen and O_3_, and non-environmental factors, such as additional rice cultivars, on the variations of the model parameters in photosynthesis and stomatal conductance models and the further impacts on simulations in large scale carbon and water cycles.

## Data Availability Statement

The raw data supporting the conclusions of this article will be made available by the authors, without undue reservation.

## Author Contributions

DW conceived the idea and led the study. YM and YC designed the experiment. YM, YC, and HW measured all the data used for this study. YM analyzed the data and wrote the manuscript with the critical suggestions by DW. All authors contributed to the article and approved the submitted version.

## Conflict of Interest

The authors declare that the research was conducted in the absence of any commercial or financial relationships that could be construed as a potential conflict of interest.

## Footnotes

1 http://www.r-project.org
