# Supplementary material for: Diurnal and Seasonal Variations in the Photosynthetic Characteristics and the Gas Exchange Simulations of Two Rice Cultivars Grown at Ambient and Elevated CO2
Source: Front Plant Sci. 2021 Apr 6;12:651606. doi: 10.3389/fpls.2021.651606 (PMC8056013; doi:10.3389/fpls.2021.651606)
Supplement: Supplementary file 1 [file Data_Sheet_1.docx]

Supplementary Materials

# Supplementary Figures and Tables


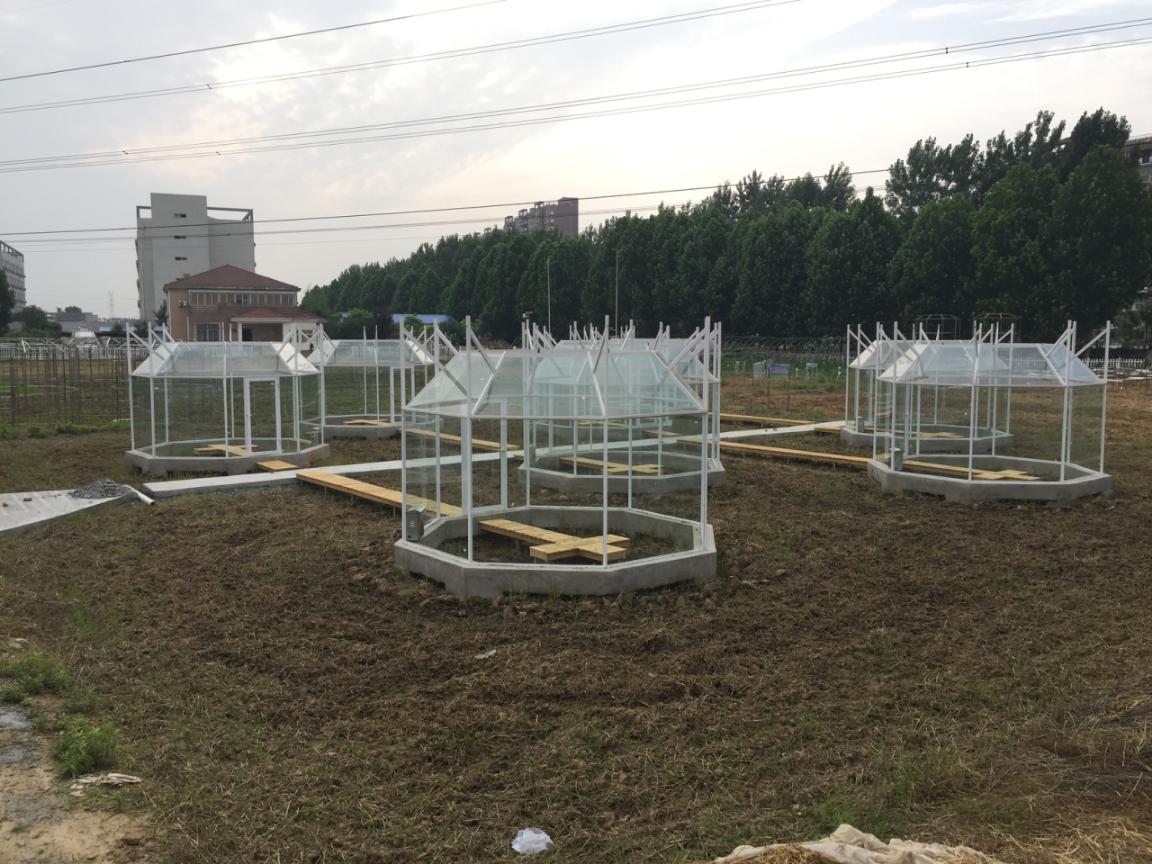


**(A)**


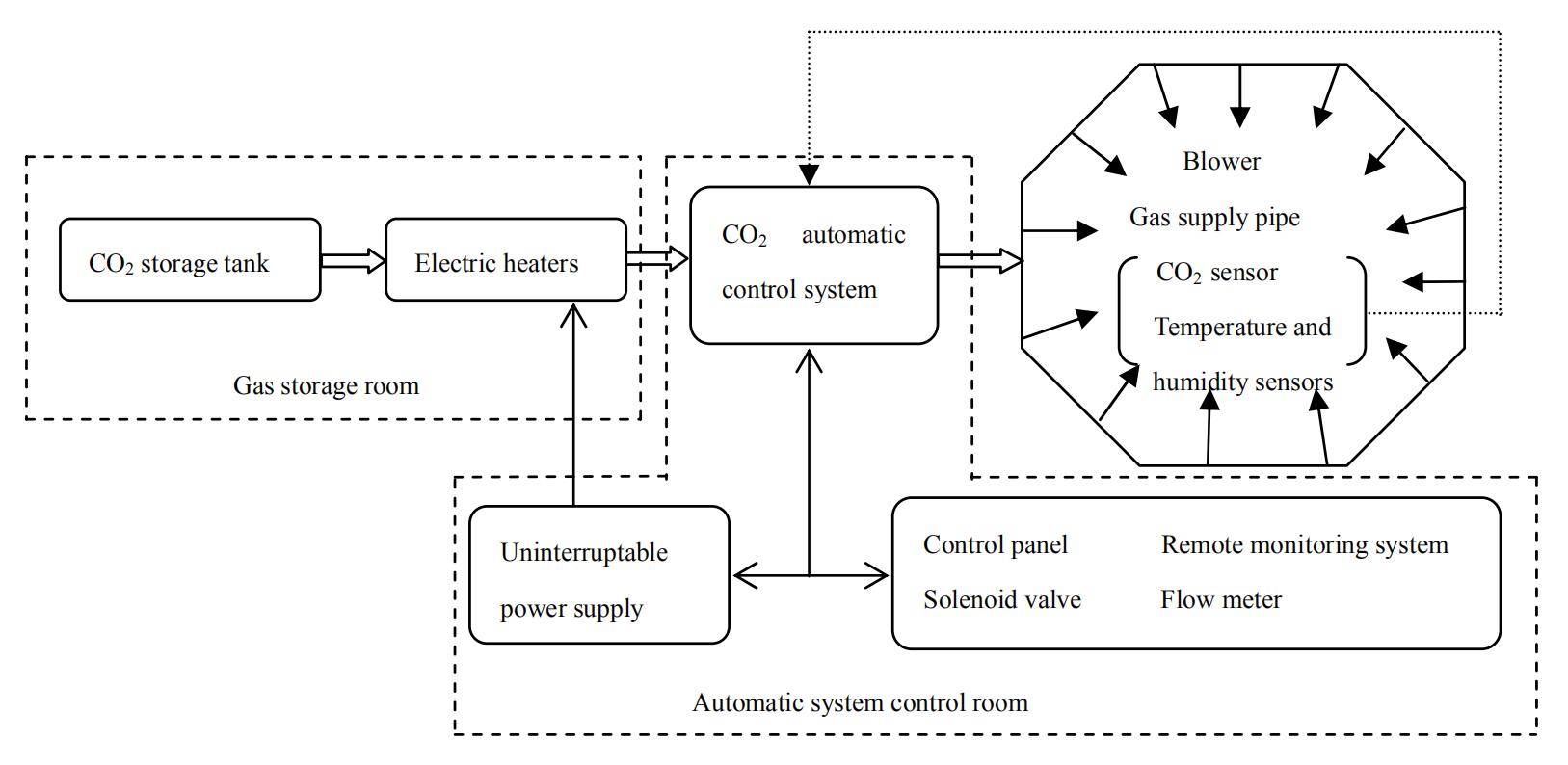


**(B)**

**S 1.** (A) Agrometeorological experimental station in Nanjing University of Information Science & Technology, in Nanjing, Jiangsu province of China (32°16′N, 118°86′E). (B) Illustration of CO_2_ Control System. Liquid CO_2_ is stored in CO_2_ storage tanks and will flow into the chambers with the control of the gas automatic control system. Electric heaters will help with the vaporization of the liquid CO_2_. [CO_2_] in chambers can be adjusted by the control panel and monitored through the remote monitoring system.


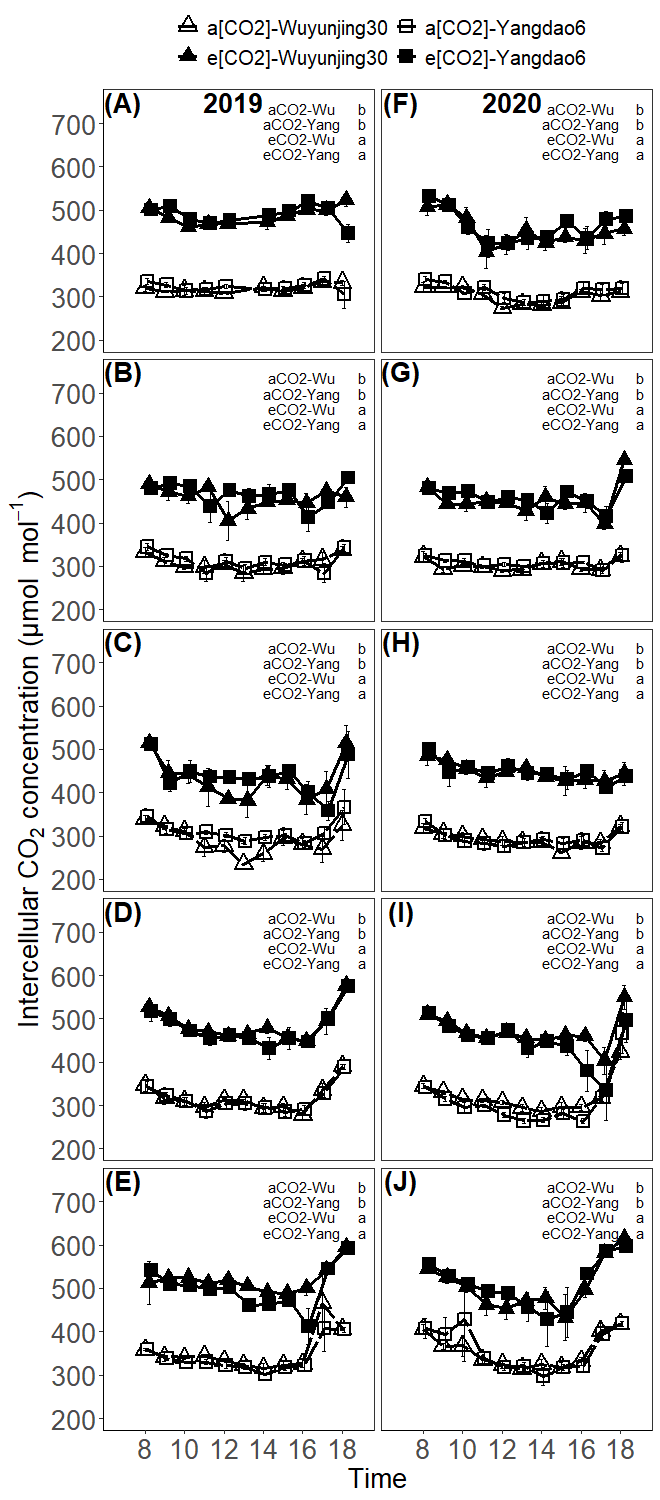


**S 2.** Diurnal variation of intercellular CO_2_ concentration (C_i_) of rice cultivar Wuyunjing30 and Yangdao6 grown under ambient and elevated [CO_2_] at jointing (A, F), booting (B, G), heading (C, H), grain-filling (D, I) and maturity (E, J) stages in 2019 and 2020. Values were expressed as means ± standard errors form. Values of C_i_ under four [CO_2_]×cultivar treatments at 13 p.m. at jointing stage in 2019 were deleted because of the abnormal values found by examination. Statistical analyses of multiple comparisons for five growing stages in two years were shown in each panels of the figure in the form of lowercase letters.


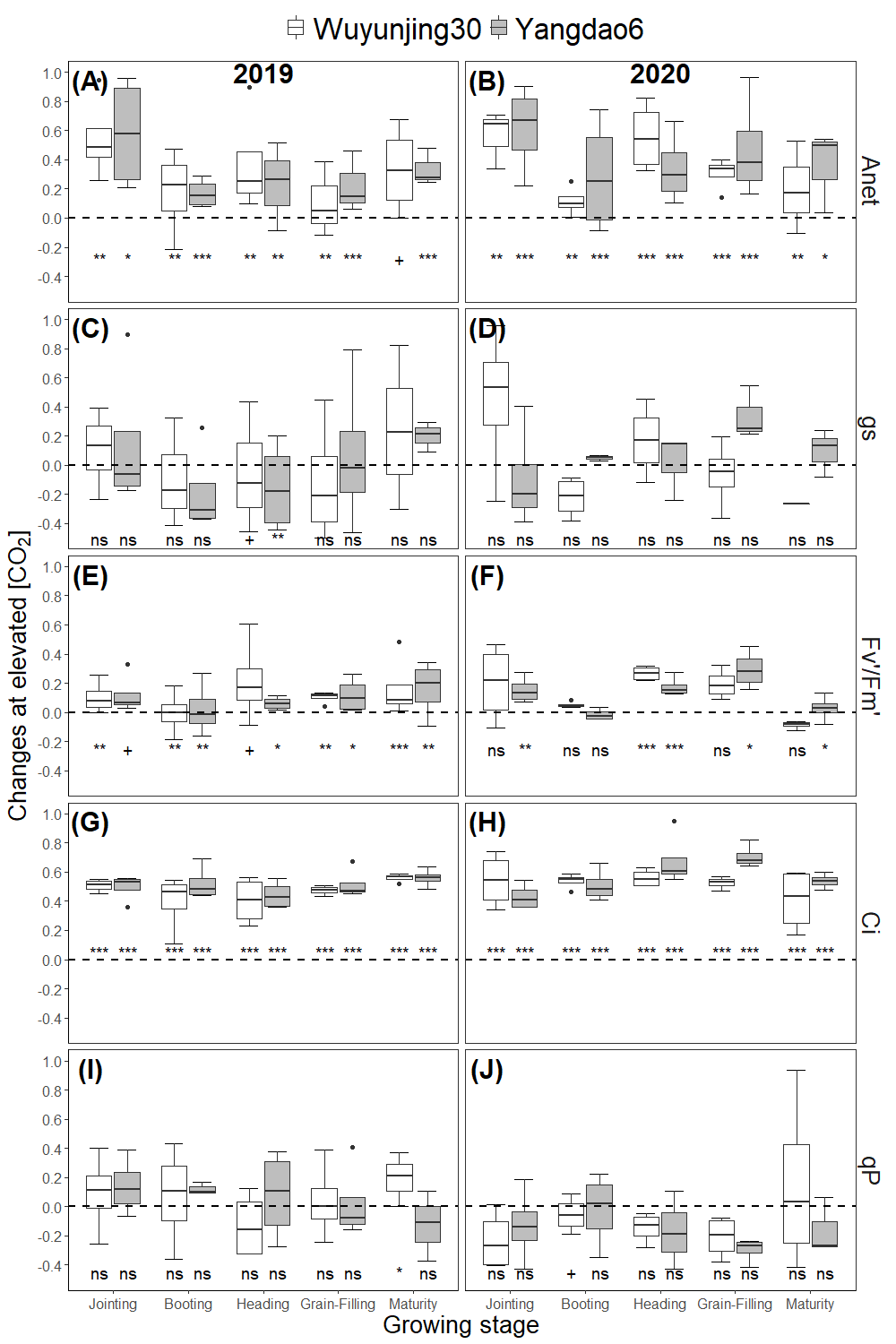


**S 3.** Responses of photosynthetic characteristics of two rice cultivars to elevated [CO_2_] at five growing stages in 2019 and 2020. Data were selected from diurnal variation data measured at 12 p.m. for less CO_2_-responsive cultivar Wuyunjing30 (white boxes) and more CO_2_-responsive cultivar Yangdao6 (grey boxes). Anet, net photosynthetic rate; gs, stomatal conductance; Fv’/Fm’, photochemical efficiency of PSⅡ; Ci, intercellular CO_2_ concentration; qP, photochemical quenching. ANOVA analyses for each photosynthetic characteristic of two cultivars at each growing stage were given in the plot. Notes: ***: Pr(>F) <0.001; **: Pr(>F) <0.01; *: Pr(>F) <0.05; +: Pr(>F) <0.1; ns: not significant.


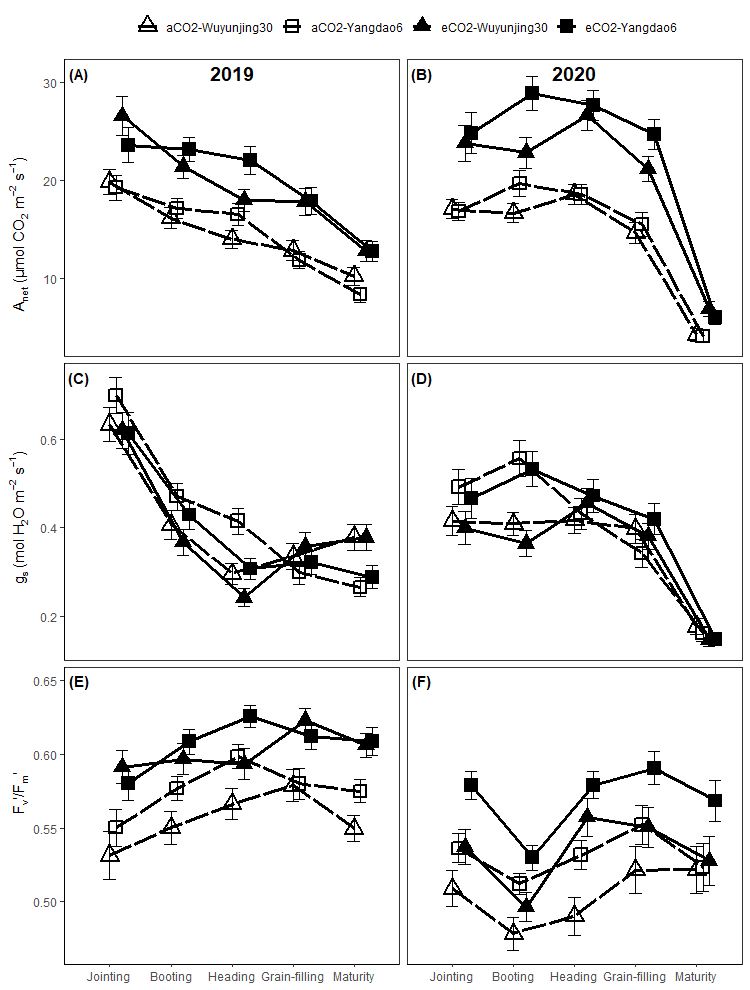


**S 4.** Seasonal variation of net photosynthetic rate (A, B), stomatal conductance (C, D) and photochemical efficiency of PSⅡ (E, F) of two rice cultivars grown under ambient and elevated [CO_2_] in 2019 and 2020. Values are expressed as means ± standard errors form.


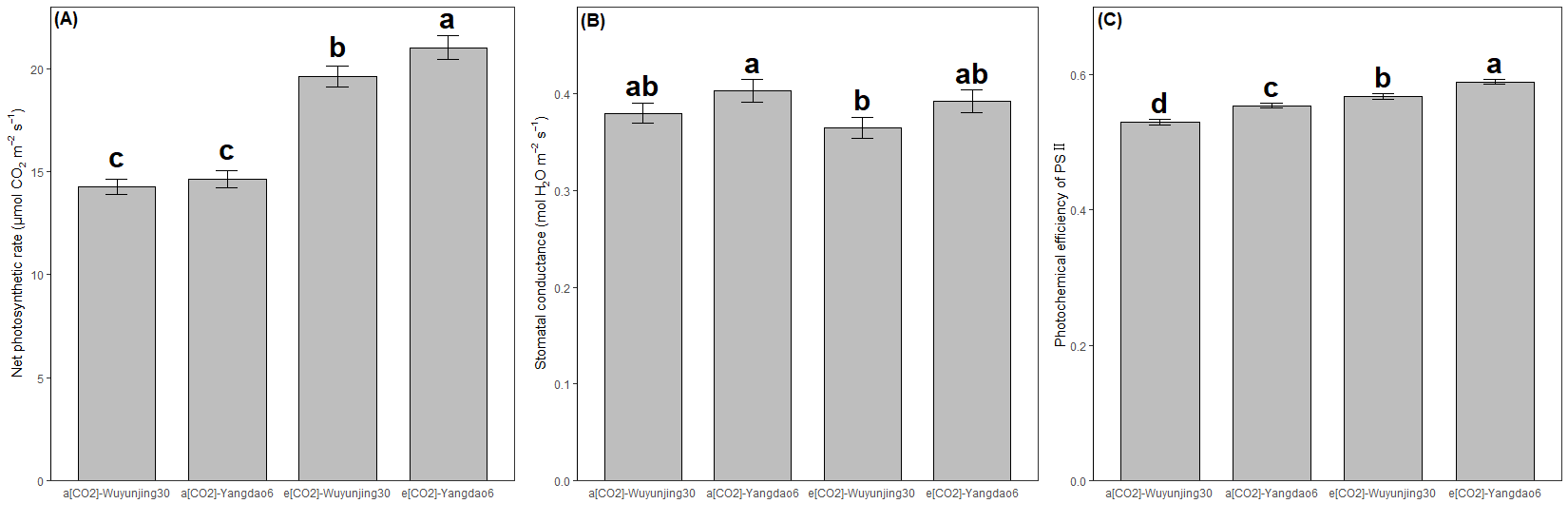


**S 5.** Averaged net photosynthetic rate (A), stomatal conductance (B) and photochemical efficiency of PSⅡ (C) of rice cultivar Wuyunjing30 and Yangdao6 grown under ambient and elevated [CO_2_] across two growing seasons. Values are expressed as means ± standard errors form. Results of the multiple comparisons are marked in the form of lowercase letters.


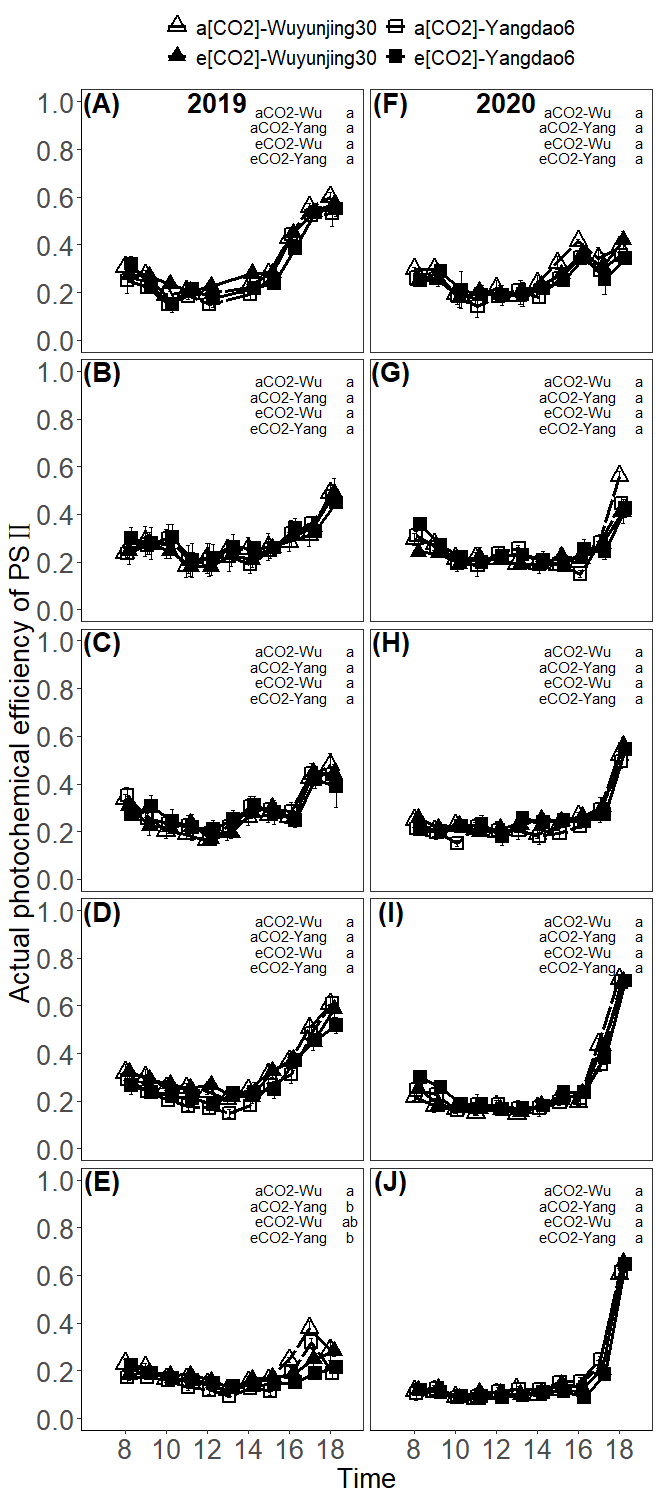


**S 6.** Diurnal variation of actual photochemical efficiency (ΦPSⅡ) of rice cultivar Wuyunjing30 and Yangdao6 grown under ambient and elevated [CO_2_] at jointing (A, F), booting (B, G), heading (C, H), grain-filling (D, I) and maturity (E, J) stages in 2019 and 2020. Values were expressed as means ± standard errors form. Values of ΦPSⅡ under four [CO_2_]×cultivar treatments at 13 p.m. at jointing stage in 2019 were deleted because of the abnormal values found by examination. Statistical analyses of multiple comparisons for five growing stages in two years were shown in each panels of the figure in the form of lowercase letters.


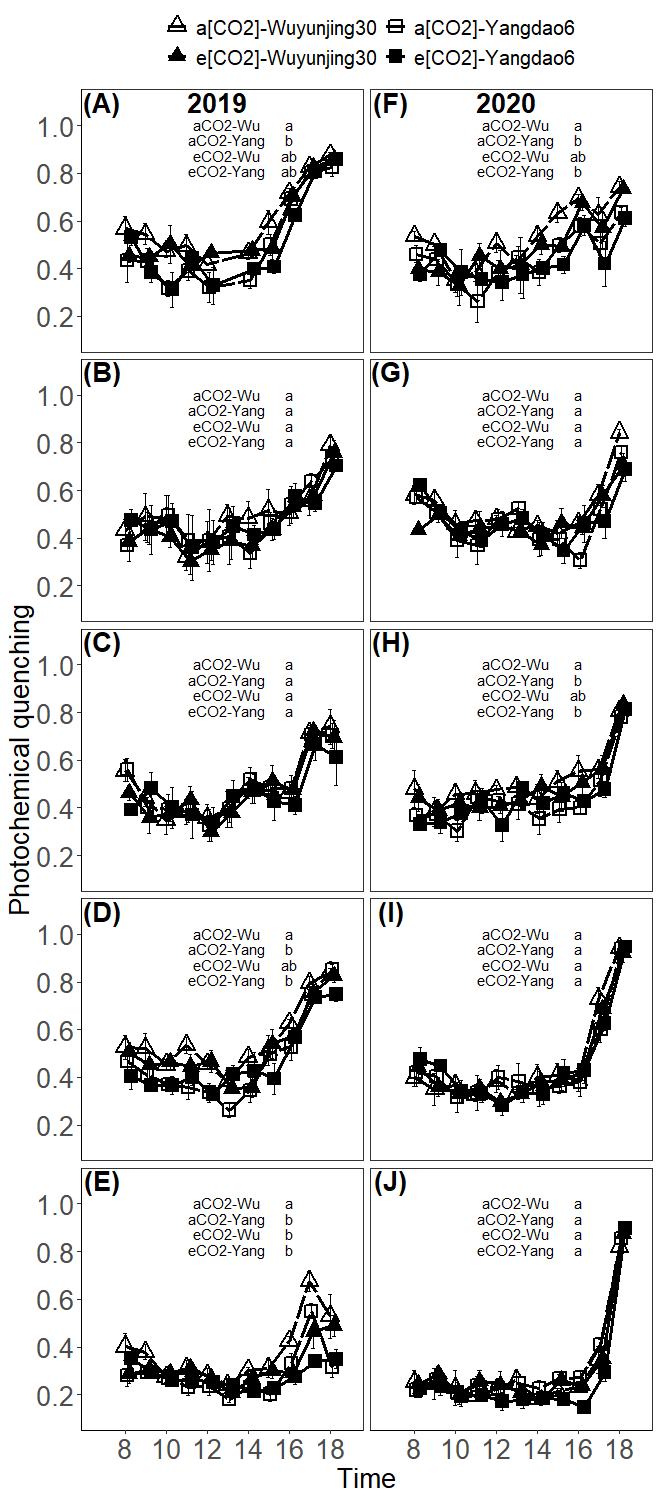


**S 7.** Diurnal variation of photochemical quenching (qP) of rice cultivar Wuyunjing30 and Yangdao6 grown under ambient and elevated [CO_2_] at jointing (A, F), booting (B, G), heading (C, H), grain-filling (D, I) and maturity (E, J) stages in 2019 and 2020. Values were expressed as means ± standard errors form. Values of qP under four [CO_2_]×cultivar treatments at 13 p.m. at jointing stage in 2019 were deleted because of the abnormal values found by examination. Statistical analyses of multiple comparisons for five growing stages in two years were shown in each panels of the figure in the form of lowercase letters.


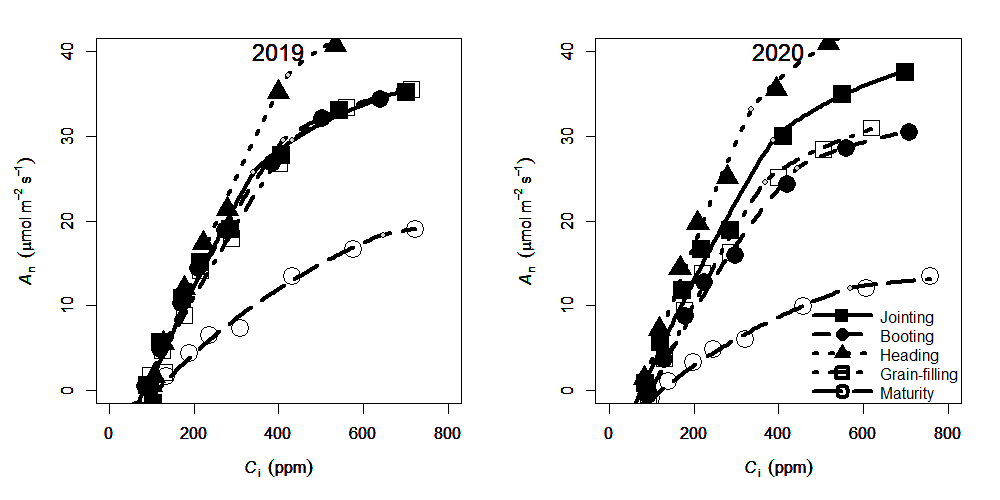


**S 8.** A_net_-Ci curves of rice leaves measured at jointing (black square), booting (black circle), heading (black triangle), grain-filling (white square) and maturity stage (white circle) in 2019 and 2020. Values have been averaged over four [CO2]×cultivar treatments.


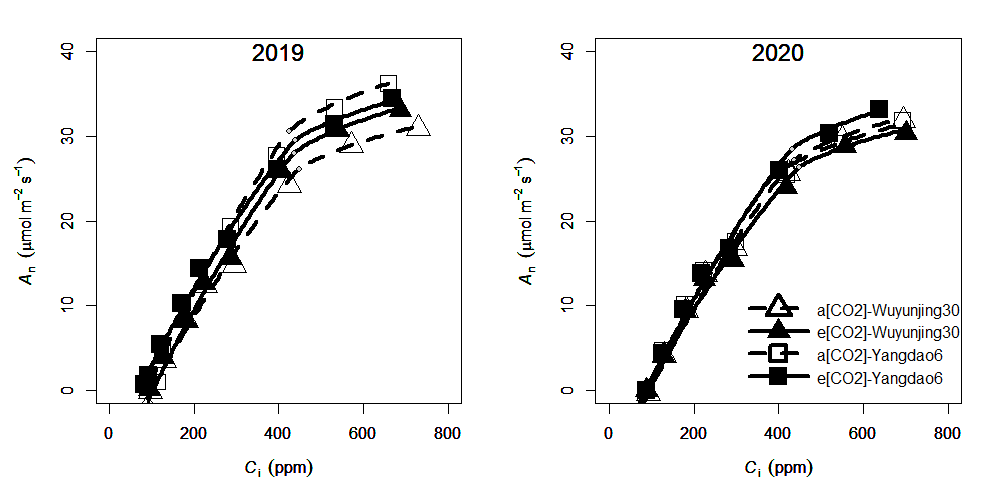


**S 9.** A_net_-Ci curves of rice leaves measured at five growing stages in 2019 and 2020 for Wuyunjing30 grown under ambient (white triangle) and elevated [CO_2_] (black triangle), and Yangdao6 grown under ambient (white square) and elevated [CO_2_] (black square). Values have been averaged over five growing stages.
